# Insights from Cardiopulmonary Exercise Testing in Pediatric Patients with Hypertrophic Cardiomyopathy

**DOI:** 10.3390/biom11030376

**Published:** 2021-03-02

**Authors:** Giovanna Gallo, Vittoria Mastromarino, Giuseppe Limongelli, Giulio Calcagni, Antonello Maruotti, Luca Ragni, Fabio Valente, Maria Beatrice Musumeci, Rachele Adorisio, Marta Rubino, Camillo Autore, Damiano Magrì

**Affiliations:** 1 Department of Clinical and Molecular Medicine, Sapienza University, 00189 Rome, Italy; giovanna.gallo@uniroma1.it (G.G.); beatrice.musumeci@uniroma1.it (M.B.M.); camillo.autore@uniroma1.it (C.A.); 2 Paediatric Cardiology and ACHD Unit, S. Orsola, Malpighi Hospital, 40138 Bologna, Italy; vitti.lu@hotmail.it (V.M.); luca.ragni@aosp.bo.it (L.R.); 3 Cardiologia SUN, Monaldi Hospital, II University of Naples, 80100 Naples, Italy; giuseppe.limongelli@libero.it (G.L.); dr.valentefabio@gmail.com (F.V.); rubinomarta@libero.it (M.R.); 4 Department of Pediatric Cardiology and Cardiac Surgery, Bambino Gesù Children’s Hospital, IRCCS, 00050 Rome, Italy; giulio.calcagni@opbg.net (G.C.); rachele.adorisio@opbg.net (R.A.); 5 Department of Scienze Economiche, Politiche e delle Lingue Moderne, Libera Università SS Maria Assunta, 00193 Rome, Italy; a.maruotti@lumsa.it; 6 Department of Mathematics, University of Bergen, 5052 Bergen, Norway; 7 School of Computing, University of Portsmouth, Portsmouth PO2 8QD, UK

**Keywords:** hypertrophic cardiomyopathy, pediatric, clinical assessment, cardiopulmonary exercise test

## Abstract

The usefulness of cardiopulmonary exercise test (CPET) in adult hypertrophic cardiomyopathy (HCM) patients is well-known, whereas its role in pediatric HCM patients has not yet been explored. The present study investigates possible insights from a CPET assessment in a cohort of pediatric HCM outpatients in terms of functional and prognostic assessment. Sixty consecutive pediatric HCM outpatients aged <18 years old were enrolled, each of them undergoing a full clinical assessment including a CPET; a group of 60 healthy subjects served as controls. A unique composite end-point of heart failure (HF) related and sudden cardiac death (SCD) or SCD-equivalent events was also explored. During a median follow-up of 53 months (25th–75th: 13–84 months), a total of 13 HF- and 7 SCD-related first events were collected. Compared to controls, HCM patients showed an impaired functional capacity with most of them showing peak oxygen uptake (pVO_2_) values of <80% of the predicted, clearly discrepant with functional New York Heart Association class assessment. The composite end-point occurred more frequently in patients with the worst CPETs’ profiles. At the univariate analysis, pVO_2_% was the variable with the strongest association with adverse events at follow-up (C-index = 0.72, *p* = 0.025) and a cut-off value equal to 60% was the most accurate in identifying those patients at the highest risk. In a pediatric HCM subset, the CPET assessment allows a true functional capacity estimation and it might be helpful in identifying early those patients at high risk of events.

## 1. Introduction

Hypertrophic cardiomyopathy (HCM), the most common genetic heart disease, inherited with an autosomal dominant pattern, incomplete penetrance, and variable expressivity, is characterized by extremely varied phenotypic expression ranging from asymptomatic to heart failure (HF) to sudden cardiac death (SCD) [1]. In such a context, notwithstanding children with HCM are considered in the highest risk spectrum [2,3,4], the most common recommendations on pharmacological and non-pharmacological treatment (i.e., drugs, implantable cardioverter defibrillator, ICD, septal reduction procedures, inclusion in cardiac transplantation list, etc.), are often disregarded or too much postponed in this setting most likely because of possible detrimental effects of some strategies on patients’ quality of life (i.e., drugs’ side effects, ICDs inappropriate shocks or failure, depression, etc.) [3,4,5,6]. However, another reason underlying this conservative approach is undoubtedly the highly variable risk perception among physicians due to the lack of strong evidence-based risk prediction models in childhood HCM [7]. Accordingly, besides the historical markers of disease severity [8,9], it is conceivable that, properly in young HCM patients, an adjunctive analysis of the exercise capacity by means of a cardiopulmonary exercise test (CPET) could be extremely useful with respect to their clinical management. Indeed, growing evidence suggests that a full CPET assessment, in combination with other clinical and instrumental variables, is able to stratify both the SCD and the HF risk in adult cohort of HCM patients [10,11,12,13,14,15]. Furthermore, regardless its potential prognostic value in pediatric HCM patients, it is likely that a systematic CPET assessment might be helpful in this setting to disclose an unsuspected functional limitation [16,17], to develop individualized exercise training programs to prevent deconditioning [18,19], and, last but not least, to instill confidence in young HCM patients and their relatives through a maximal exercise test performed without side effects [20,21].

Therefore, the actual multicenter retrospective study investigates possible clinical insights coming from a full CPET assessment in a cohort of pediatric HCM outpatients aged less than 18 years old. First, we explored the CPET data in our study cohort and in a healthy control group matched for general characteristics as well as we analyzed critically the prevalence of a functional impairment in terms of peak oxygen uptake (pVO_2_) with respect to the one assessed merely in terms of New York Heart Association (NYHA). Thereafter, we sought to analyze possible association between a number of CPET-derived variables and composite cardiovascular end-point, including HF-related events and SCD or SCD-equivalent events.

## 2. Materials and Methods

### 2.1. Patients’ Study Sample

The initial study cohort consisted of 66 consecutive pediatric outpatients with HCM aged <18 years-old (age range of 10–17 years); they were recruited and followed in 4 HCM Italian centers between September 2014 and September 2021: Sant’Andrea Hospital, Sapienza University, Rome (*n* = 40); Bambino Gesù Children’ Hospital, IRCCS (*n* = 8), Rome Monaldi Hospital, Naples (*n* = 10); and Sant’Orsola Hospital, Bologna (*n* = 8). Diagnosis of HCM was based on the presence of maximal wall thickness of ≥15 mm or greater than two standard deviations (SDs) above the body surface area-corrected population mean (Z-score ≥ 2) that could not be explained solely by abnormal loading conditions or in accordance with published criteria for the diagnosis of disease in relatives of patients with unequivocal disease [1,8,9,22].

Primary study inclusion criteria were stable clinical conditions with unchanged medications for at least 6 months and capability to perform a maximal, symptom-limited CPET. *A priori* exclusion criteria were previous septal reduction therapy and pacemaker-dependent atrial rhythm. Patients with known metabolic diseases or syndromic causes of HCM were also excluded ab initio from the analysis, and specifically, we excluded ab initio three patients affected by Fabry Disease, two with Noonan Syndrome, and three with Noonan Syndrome Multiple Lentigines Syndrome.

Data were independently collected at each participating center using a uniform methodology. The study complied with the ethical standards of the Declaration of Helsinki and was reviewed and approved by the institutional ethics committee. Written informed consent was given by all participants.

### 2.2. Patients’ Clinical Assessment

Each HCM patient who fulfilled the initial inclusion criteria underwent a full clinical assessment, including clinical history with pedigree analysis and NYHA classification, 24 h ECG Holter monitoring, transthoracic Doppler echocardiography, and CPET. The usual five SCD risk factors were also collected [8]: (a) familiar history of SCD (history of HCM-related SCD in at least 1 first-degree or other relatives who were <50 years old); (b) massive left ventricular (LV) hypertrophy (maximal wall thickness, MWT, ≥30 mm) or Z-score ≥ 6); (c) at least 1 run of non-sustained ventricular tachycardia (≥3 consecutive ventricular beats at ≥120 beats/min and <30 s in duration on 24 h ECG Holter monitoring); (d) unexplained syncope judged inconsistent with neurocardiogenic origin; and (e) abnormal blood pressure response to exercise (ABPRE, failure to increase systolic blood pressure by at least 20 mmHg from rest to peak exercise or a fall of ≥20 mmHg). The following echocardiographic measurements, obtained according to international guidelines [22,23], were considered: LV end-diastolic diameter (parasternal long axis); the greatest LV thickness (maximal wall thickness measured at any LV site); left atrial diameter (LAd, parasternal long axis); the highest maximal LV outflow tract gradient among those measured at rest, in the orthostatic position, and after the Valsalva maneuver (LVOTG_max_, apical 4-chamber view) [22,23], and LV ejection fraction (LVEF) with Simpson’s biplane methods (LVEF, apical 4-chamber view). To account for somatic growth, MWT and LAd were expressed both in millimeters and as Z-scores defined as the SDs’ number from the population mean [24].

A maximal, symptom-limited CPET was performed on an electronically braked cycloergometer, and a personalized ramp exercise protocol was chosen, aiming at a test duration of 10 ± 2 min [25]. The exercise was preceded by a few minutes of resting breath-by-breath gas exchange monitoring and by a 3 min unloaded warm-up. CPET was self-terminated by the subjects when they claimed that they had achieved maximal effort but it was considered truly maximal or nearly maximal if the respiratory exchange ratio (R.E.R.) was ≥1.05. A 12-lead ECG, diastolic blood pressure, and systolic blood pressure (SBP) were recorded during CPET. Baseline heart rate (HR) and peak HR (pHR) were also collected during CPETs; baseline HR being measured after at least 2 min of rest in a seated position on the cycloergometer. Peak HR was also analyzed as a percentage of the maximum predicted values according to the standard formula [26]:

%pHR = {[pHR/(220 − age)] × 100}.(1)

A breath-by-breath analysis of expiratory gases and ventilation (VE) has been performed, and peak values were obtained in the last 20 s of exercise. The predicted peak VO_2_ (pVO_2_) was determined using the gender-, age-, and weight-adjusted Hansen/Wasserman equations [27]. Circulatory power (CP = pVO_2_, mL/kg/min * SBP, mmHg) was obtained also considering pVO_2_ value as percentage of predicted (CP%) [13,15,17,28]. Anaerobic threshold (AT) was measured by V-slope analysis of VO_2_ and carbon dioxide production (VCO_2_), and it was confirmed by ventilator equivalents and end-tidal pressures of CO_2_ and O_2_. The end of the isocapnic buffering period was identified when VE/VCO_2_ increased and end-tidal pressure of CO_2_ decreased. VE/VCO_2_ slope was calculated as the slope of the linear relationship between VE and VCO_2_ from the first minute after the beginning of the loaded exercise and the end of the isocapnic buffering period [27].

Of note, due to the study sample’s specificity, we also considered the Z-score values for pVO_2_ and VE/VCO_2_ slope as recently proposed by Blanchard and colleagues [29].

### 2.3. Study End-Point

All patients had planned clinical reviews every 6–12 months or earlier according to their clinical status. Follow-up duration was defined as the time interval between the CPET examination and either the first event or the last visit/telephone interview in the case of no events.

A unique composite cardiovascular end-point was tested, including both HF-related events and SCD or SCD-equivalent events. The HF-related events included the following: death from HF, cardiac transplantation, progression to NYHA class III–IV caused by end-stage phase with or without LVEF < 50% (hypokinetic dilated phase or restrictive phenotype evolution), hospitalization because of development of HF symptoms or signs, and septal reduction procedure for development of significant HF signs/symptoms. The SCD and SCD-equivalent events included: SCD events, aborted SCD, and appropriate ICD shock on ventricular fibrillation or sustained ventricular tachycardia. Death from other causes or other cardiovascular events (i.e., atrial fibrillation occurrence) was not considered in the present survival analysis to avoid an excessive heterogeneity within the composite outcome. The causes of death, as well as the other events, were ascertained by experienced cardiologists at each center using hospital and primary healthcare records, death certificates, post-mortem reports, and interviews with relatives and/or physicians.

### 2.4. Statistical Analysis

Unless otherwise indicated, all data are expressed as mean ± SD. Categorical variables were compared with a difference between proportion tests; a two-sample *t* test was used to compare the continuous data between groups. Preliminarily, an extension of the Shapiro–-Wilk test of normality was performed. We therefore focused on the distribution of the survival times by adopting the Cox proportional hazards regression model. The univariate survival analysis was performed by adopting the stratified Cox regression model, allowing for separate baseline functions for each stratum according to the beta-blocker use. Discrimination of variables to be included in a possible final multivariate model was performed by C-index. We retained the models with the best trade-off between model complexity and model fit judged by the log-likelihood (using the Akaike Information Criterion). To determine whether a fitted Cox regression model adequately describes the data, we considered three kinds of diagnostics, i.e., (i) for violation of the assumption of proportional hazards, (ii) for influential data, and (iii) for non-linearity in the relationship between the log hazard and the predictors. A test of the proportional hazards assumption was performed for each covariate by correlating the corresponding set of scaled Schoenfeld residuals with a transformation of time based on the Kaplan–Meier estimate of the survival function. Last, a receiver operating characteristic (ROC) analysis has been used to determine the predictive capability of specific variables in identifying the pre-specified end-point. The behavior of a cut-off-dependent performance measure, such as accuracy, was considered across the range of all cut-offs. Cut-off values were identified maximizing the accuracy ((true positive + true negative)/total sample). The optimal cut-off was the threshold that maximized the distance to the identity (diagonal) line according to Youden’s J statistic. The cut-off values were accordingly tested in the univariate survival analysis. Statistical analysis was performed using R (R Development Core Team, 2014). A *p*-value lower than or equal to 0.05 was generally considered as statistically significant.

## 3. Results

Starting from a total sample of 66 consecutive pediatric HCM outpatients, 6 patients were excluded because of the presence of one or more of the following criteria: CPET data not fully interpretable (*n* = 5), metabolic maximal effort (respiratory exchange ratio < 1.05) not achieved (*n* = 2), and previous septal reduction therapy (*n* = 1). Notably, no major adverse events (i.e., arrhythmias) were observed during the CPET execution. A total of 60 pediatric HCM patients on optimized therapy met the inclusion criteria and were, therefore, considered as suitable for the study. CPETs data from 60 healthy young subjects, matched for age (15 ± 2 years vs. 14 ± 2 years), gender (male 73% vs. 75%), and BSA (1.7 ± 0.4 m^2^ vs. 1.6 ± 0.3 m^2^), were resumed and re-analyzed to create a healthy control group with respect the CPETs data.

### 3.1. General Characteristics of the Study Population

All the demographic and clinical characteristics of the HCM cohort are reported in Table 1. The population mainly consisted of male patients (*n* = 44 patients, 73%) in NYHA I functional class (*n* = 45 patients, 75%); 11 patients (18%) had Doppler evidence of left ventricular outflow tract (LVOT) obstruction (LVOT gradient ranging between 30 to 85 mm Hg), and 12 patients (20%) had been implanted with an ICD. During the entire follow-up, further 14 (23%) additional patients underwent ICD implantation. No documented cardiovascular comorbidities have been disclosed during the clinical assessment. Pharmacological treatment comprised beta-blocker (*n* = 35 patients, 58%), verapamil (*n* = 1 patients, 2%), angiotensin converting enzyme inhibitors or angiotensin-receptor blockers (*n* = 3 patients, 5%), and diuretics (*n* = 4 patients, 7%).

**Figure 1:**
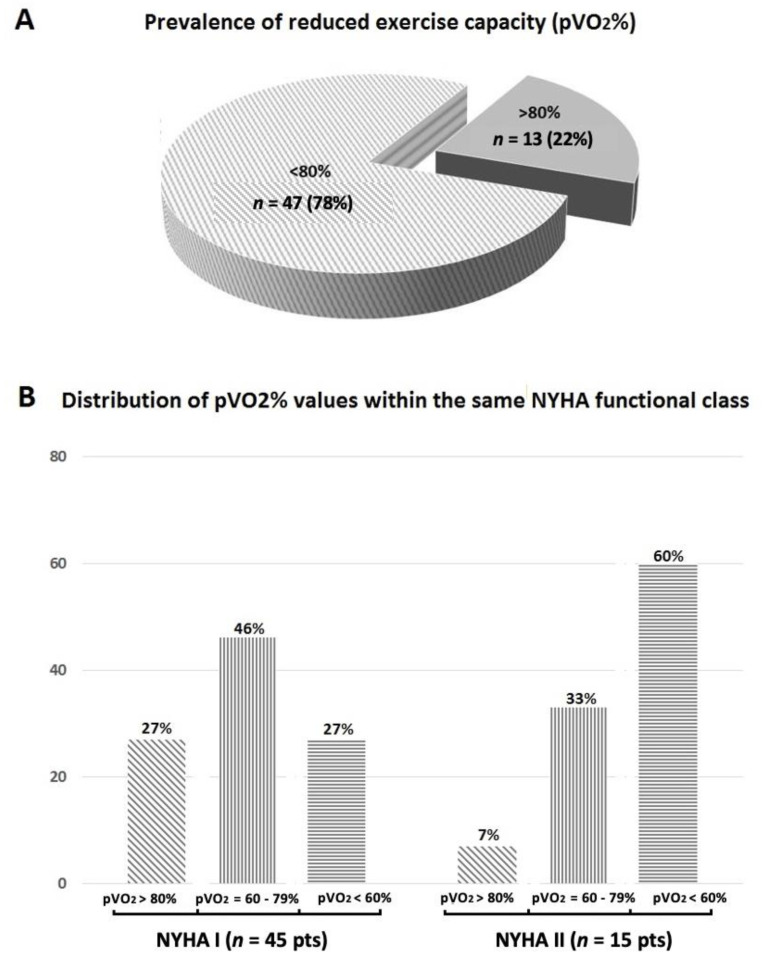
Prevalence of reduced exercise capacity, as assessed in terms of peak oxygen uptake (pVO_2_) of <80% of the maximum predicted in the entire hypertrophic cardiomyopathy (HCM) study sample (panel **A**) and dispersion of pVO_2_ values within the same New York Heart Association (NYHA) functional class (panel **B**).

An impaired exercise capacity, as assessed in terms of pVO_2_ values of <80% of the predicted, has been found in the 78% of the study sample (*n* = 47 patients) (Figure 1A). Despite most of the HCM patients were classified in NYHA I functional class, most of them (*n* = 33.73%) showed a reduced exercise capacity, the percentage of impaired exercise capacity raising in the NYHA II group (*n* = 14 patients, 93%) (Figure 1B).

**Figure 2:**
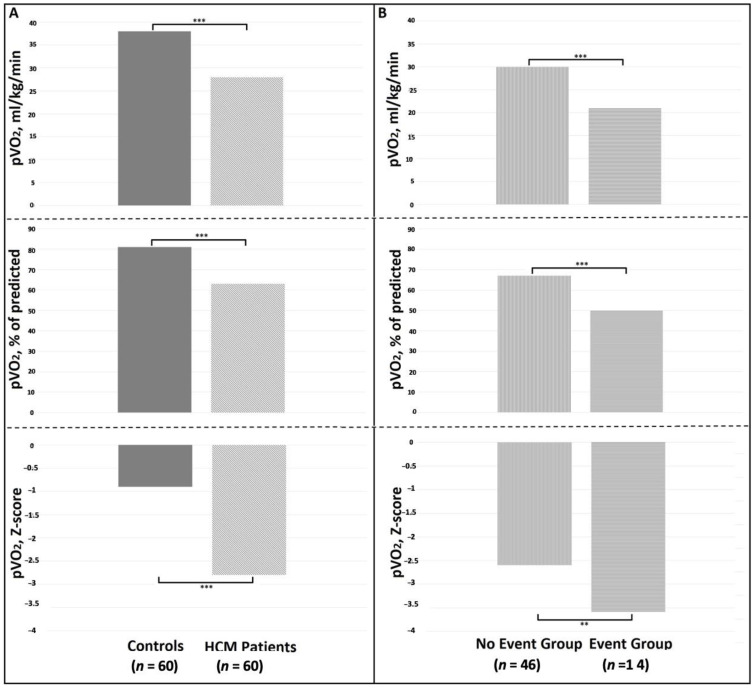
Comparison between peak oxygen uptake (pVO_2_) values between healthy controls and HCM patients (column **A**) and between HCM patients who did not experience (No Event Group) or experienced (Event Group) adverse events (column **B**). *** *p* < 0.0001; ** *p* < 0.001.

With respect to the Control Group, the HCM patients showed a significantly poorer functional status in terms of maximum workload achieved, pVO_2_ (regardless the adopted correction) (Figure 2A), CP, and VE/VCO_2_ slope values (Table 2).

Last, by analyzing the HCM sample according to the occurrence of the pre-specified cardiovascular events, those patients who experience adverse events during the follow-up (Event Group) showed higher prevalence of unexplained syncope and ABPRE, higher LAd, and lower LVEF values than the counterpart (Table 3). Noteworthy, the Event Group showed the worst CPET profile (Table 4, Figure 2B). Last, with respect the pharmacological treatment, no difference was found between groups for any medications.

### 3.2. Survival Analysis

During a median follow-up of 53 months (25th–75th percentile: 13–84 months), a total of 20 (33%) pre-specified events were collected of whom 13 were HF-related events (two heart transplantation, five evolution to an established NYHA III–IV class, three hospitalization because of HF signs/symptoms and three hospitalization for septal reduction procedure due to development of significant HF signs/symptoms) and 7 were SCD or SCD equivalent events (two SCD, two aborted SCD, and three appropriate ICD shocks). However, in patients who developed multiple events, time to the first event was used as the event time cut-off leading to a total of 14 first cardiovascular events (10 HF-related events and 4 SCD or SCD equivalents) with an estimated cumulative hazard at 10-year equal to 0.54 (95% confidence interval, 0.2–1.0). Patients who ended the follow-up period before the 10th year were considered censored at the time of the last clinical evaluation.

Many single clinical variables were associated at univariate analysis at the composite end-point, i.e., covariates with the best association being for LAd when expressed as Z-score (C-index 0.69, *p* = 0.014), pVO_2_% (C-index 0.72, *p* = 0.025), and CP% (C-index 0.71, *p* = 0.021) (Table 5). Conversely, due to the relatively low number of first cardiovascular events (*n* = 14), the composite end-point was not challenged in a multivariate model.

**Figure 3:**
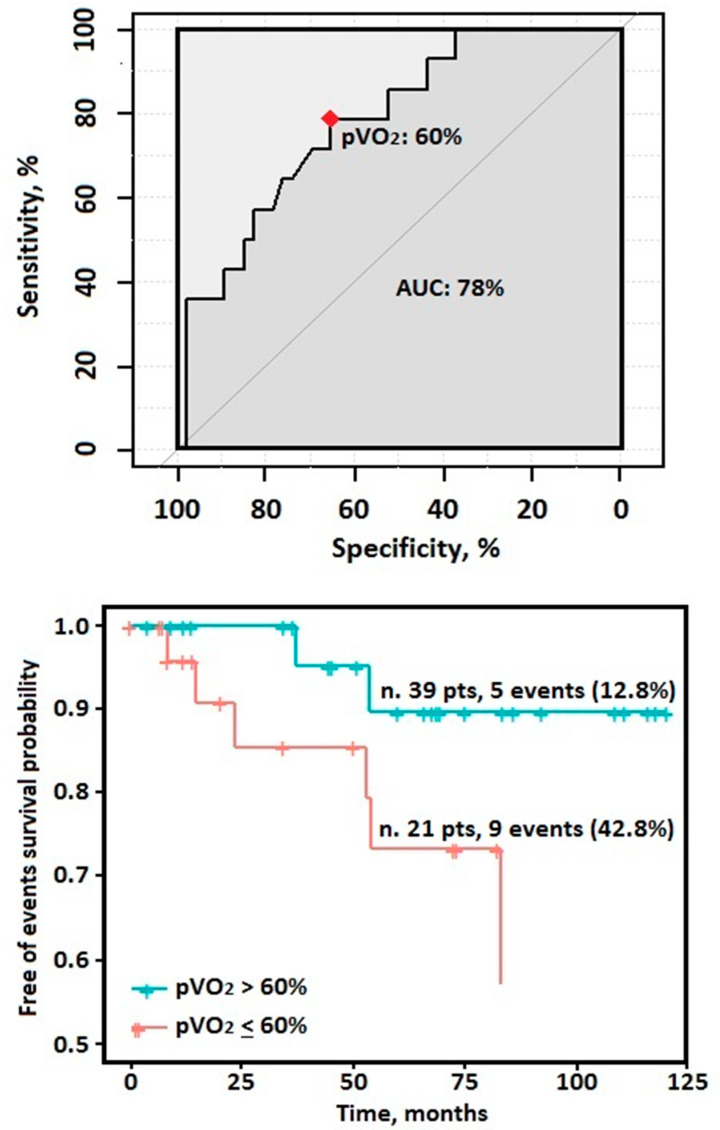
(**Upper**) Receiver-operating characteristic (ROC) analysis showing the point with the best accuracy of the peak oxygen uptake (pVO_2_) in the HCM study sample (*n* = 60). (**Lower**) Kaplan–Meier estimator of cardiovascular events for the pVO_2_ according to a 60% cutoff value.

The ROC analysis identified a pVO_2_% equal to 60% as the best cut-off value in predicting the composite end-point within the entire HCM study cohort (sensitivity: 79%; specificity: 66%; positive predictive value 98%; negative predictive value: 11%; AUC 78%) (Figure 3). Notably, a pVO_2_ values of <60% have been found in 21 patients corresponding to the 35% of the overall sample.

## 4. Discussion

The present multicenter retrospective study, conducted on a relatively small cohort of pediatric HCM outpatients < 18 years old and regularly followed at 4 Italian tertiary HCM centers, shows an exercise capacity significantly impaired in this setting of HCM patients and, contextually, it warms strongly against a reliable assessment of functional capacity by means of a mere NYHA classification. In addition, albeit obtained in a small study sample, our data argue in favor of a routine CPET assessment in pediatric HCM patients to identify those at the highest risk of future HCM-related events.

A significant portion of HCM patients reports limiting symptoms such as exertional dyspnea and fatigue [16,20,21], however an objective evaluation of these symptoms remains difficult, also considering the complex pathophysiological mechanisms possibly implied [1,16,17]. Our data, in a cohort of apparently asymptomatic or just slightly symptomatic pediatric HCM patients, raises several concerns about the NYHA classification reliability in HCM population [10,12,16]. Indeed, all the most relevant CPET-derived data collected were hugely worse than those obtained in a healthy control group matched for anthropometric characteristics. Furthermore, most of the patients classified as in NYHA I failed to achieve at least a pVO_2_ equal to the 80% and even, in more than one-third of cases, neither the 60% of the maximum predicted. Similarly, even in those patients classified as in NYHA II class, there was a huge portion with pVO_2_ values severely impaired. Thus, as it happens in other cardiac diseases in the youngness, as in complex palliated congenital heart defects or in dilated cardiomyopathy or [30,31], also in pediatric HCM patients, the NYHA classification system suffers from a lack of accuracy in ascertaining the true functional capacity. In such a context, a maximal CPET might be useful to grade objectively the exercise impairment and, possibly, to follow-up their clinical course and to guide therapeutic options. Furthermore, throughout this safe and noninvasive approach, it could be possible to establish safe exercise training programs, the latter being an emerging issue since that the most of the HCM patients have important reservation about physical activity [18,19,32,33]. Last, a CPET assessment possibly combined with a contextual echocardiographic Doppler analysis might improve the physicians’ comprehension of the complex mechanisms underlying an impaired pVO_2_ in HCM patients [17]. Indeed, besides the occurrence of the well-known disease-related complications (i.e., atrial fibrillation or end-stage phase), myocardial fiber disarray, interstitial fibrosis, microvascular ischemia, chronotropic incompetence, as well as LVOT obstruction, are all factors able to impact negatively on diastolic and systolic left ventricular function at rest and, particularly, during exercise [17]. Although each of the abovementioned clinical features might concur theoretically to an exercise impairment also in pediatric HCM patients, it might be conceivable that, particularly in this setting, the genetic burden might play the greatest role through a direct impact on the cardiomyocytes’ efficiency [15,34].

Although a usual benign course with a quite preserved life expectancy [1], the prediction of adverse events in the HCM population represents still a challenging research field and a number of variables have been investigated and variously combined to obtain optimal prognostic models [8,9,13,14]. However, solid evidences about the pediatric HCM setting are still lacking mainly because of their underrepresentation or exclusion from the HCM studies [7,35]. Indeed, besides the so-called HCM phenocopies (i.e., inborn errors of metabolism), which are rare and known to suffer from a worse prognosis [2,3,4,5], a great uncertainty burdens the clinical management of that patients diagnosed with sarcomeric HCM in the youngness. Thus, a highly variable risk perception among physicians, together with the consciousness of the possible detrimental effects of some strategies on patients’ quality of life, leads too often to conservative approach not in line with the standard recommendations [4,5,6,7]. Most recently, a few studies tried to bridge the gap of evidence in the young HCM setting even if most of them focused predominantly on the arrhythmic risk [3,36,37,38]. A recent study by Norrish G. et al. developed and validated internally a “HCM-risk Kids score” in a large HCM cohort aged 16 years or younger. Interestingly, besides the same variables included in the standard HCM Risk-SCD score (some of them converted into the corresponding Z-score value), they found that also NYHA classification discriminated the SCD risk in such population [38]. Another paper by Maurizi et al. confirmed the importance of limiting symptoms in a cohort of 100 young HCM patients (mean age 12 years old and median follow-up 9 years) where a NYHA class > I or Ross score > 2 have been identified as the strongest independent outcome predictors, in terms of both SCD- and HF-related events [3]. Last, most recently, in nearly 400 pediatric HCM patients (median age 14 years and median follow-up 5.9 years), Alashi et al. found the presence of symptoms as an important feature associated with the primary composite outcome of SCD-related events, septal reduction procedure due to HF worsening and cardiac transplantation [37]. Accordingly, it is highly reasonable that a more reliable functional classification in pediatric HCM patients might further improve also their risk stratification as it has been proven yet in adult HCM patients [10,11,12,13,14,15]. Our data support the abovementioned hypothesis given that the most important CPET-derived variable, namely, the pVO_2_, was greatly impaired in those HCM patients who experienced a cardiac event during the follow-up and univariately associated to the tested end-point. Indeed, as per the well-known Fick Law, VO_2_ represents arithmetically the resulting number of the product between cardiac output (CO = stroke volume*heart rate) and artero-venous O_2_ difference [39]. Thus, due to its composite character, a reduced pVO_2_ is able to reassume *in se* a reduced stroke volume augmentation, a concomitant chronotropic incompetence as well as a huge number of conditions able to impact negatively on the O_2_ transport and delivery [17,39]. In such a context, albeit we recognize as more correct from a pathophysiological and clinical viewpoint to consider the pVO_2_ as a continuous variable, we also derived a cut-off value equal to the 60% of the maximum predicted as the most accurate in identifying a pediatric HCM subgroup at the highest cardiac risk (42.8% Versus 12.8%). In addition, the CP, one of the best surrogates of cardiac power [28], remained significantly associated to the study end-point. The reason underlying is easily understandable given that, due to its formula, it magnifies the clinical and prognostic power of pVO_2_ and of ABPRE, the latter depending on the intrinsic myocardial function/geometry and on peripheral autonomic reflexes usually impaired in HCM patients [13,40]. Last, we showed VE/VCO_2_ slope values significantly higher in the HCM group than in the healthy control group and even worse in those HCM patients who suffered from a cardiac event. Indeed, in HCM patients, the ventilatory efficiency is thought to mirror the diastolic dysfunction degree as well as the exercise-induced ventilation/perfusion mismatch derangement [41], and it has been demonstrated as the CPET-derived variable with the strongest association with the SCD risk in an adulthood HCM cohort [15]. However, most likely due to the small sample and the low number of arrhythmic events collected, this fashion parameter failed to achieve a statistical significance at the univariate analysis.

## 5. Limitations

The relatively small number of pediatric patients enrolled, together with the low number of hard events, represents a certain limitation that does not allow us to define the true weight of the CPET analysis in terms of cardiovascular risk prediction (i.e., we did not perform a multivariate survival analysis). Nevertheless, our findings call just for a more reliable and comprehensive functional assessment in the pediatric HCM patients’ management rather than they argue against the overall importance of the other diagnostic techniques, such as clinical history, echocardiographic variables, and genetic testing analysis. In such a context, we do not discuss about the importance of the molecular analysis of HCM genes that remains pivotal to distinguish the HCM phenocopies from the sarcomeric one as well as to identify phenotypically unaffected mutated relatives from HCM probands [8,9,42]. Furthermore, albeit still a debated topic, it has been suggested that those young HCM patients carrying variants in thin filaments genes might show a significant arrhythmic risk [3,34].

Another limitation is that we examined the prognostic effect of several clinical and instrumental variables at a single time point. Thus, we cannot exclude that changes in some variables altered our survival analysis results. Furthermore, it is highly reasonable that seriate CPET assessment, mainly in those young HCM patients at the highest risk according to the historical marker of disease’s severity, could further magnify its usefulness rather than rebut it.

Last, given that a reliable exercise test is difficult to be performed adequately in HCM children aged less than 10 years, our results would remain true in a specific pediatric subset able to perform a maximal symptom-limited CPET.

## 6. Conclusions

Our findings support the emerging literature identifying the CPET analysis as an insightful approach in the HCM clinical management, in the young HCM subset too. Indeed, in a group of young asymptomatic or slightly symptomatic HCM patients according to the NYHA system classification, the CPET allowed us to estimate accurately their functional capacity and to disclose a portion of unrecognized exercise impairment. Furthermore, our data argue in favor of a possible role of some CPET-derived variables in the early identification of those young HCM patients at highest risk of HCM-related events.

## Figures and Tables

**Table 1:** Main clinical variables of the hypertrophic cardiomyopathy (HCM) Group at the study run-in (N: 60 patients).

General Data	
Age, years	15 ± 2
BSA, m2	1.7 ± 0.4
Male, %	44 (73)
Age at diagnosis, years	10 ± 5
NYHA II, n (%)	15 (25)
LVOT obstruction, n (%)	11 (18.3)
ICD, n (%)	12 (20)
***SCD risk factors***	
NSVT, n (%)	6 (10)
FH-SCD, n (%)	3 (5)
MWT > 30 mm or Z score > 6, n (%)	12 (20)
Unexplained syncope, n (%)	8 (13)
ABPRE, n (%)	18 (30)
**Echocardiographic Data**	
LVEDd, mm	42 ± 5
LAd, mm	37 ± 8
LAd (Z-score)	2.3 ± 1.5
MWT, mm	21 ± 7
MWT (Z-score)	4.6 ± 1.5
LVOTGmax, mm Hg	10 [10]
LVEF, %	62 ± 6

Data are expressed as mean ± SD, as absolute number of patients (% on total sample) or as median [25th–75th percentile]. NYHA: New York Heart Association; ICD: implantable cardioverter defibrillator; LVOT: left ventricular outflow tract; SCD: sudden cardiac death; NSVT: non-sustained ventricular tachycardia; FH: family history; ABPRE: abnormal blood pressure response at exercise; LVEDd: left ventricular end diastolic diameter; LAd: left atrial diameter; MWT: maximum wall thickness; LVOTGmax: maximal LV outflow tract gradient; LVEF: LV ejection fraction.

**Table 2:** Comparison between the main cardiopulmonary exercise test (CPET) variables between HCM group and healthy controls.

CPET Data	HCM Group (N: 60 Patients)	Healthy Controls (N: 60 Subjects)	*p*-Values
Exercise time, minutes	10.1 ± 2.0	10.8 ± 1.7	NS
Peak workload, watts	116 ± 44	153 ± 42	<0.001
Peak SBP, mm Hg	143 ± 32	154 ± 17	0.034
Peak HR, % of predicted	78 ± 11	83 ± 7	NS
VO_2_ AT, mL/kg/min	17 ± 5	24 ± 7	<0.001
Peak VO_2_, mL/kg/min	28 ± 8	38 ± 8	<0.001
Peak VO_2_, % of predicted	63 ± 17	81 ± 13	<0.001
Peak VO_2_ (Z-score)	−2.8 ± 1.2	−0.9 ± 1.1	<0.001
CP, mL/kg/min*mm Hg	4101 ± 1840	5836 ± 1656	<0.001
CP%, % of predicted*mm Hg	9176 ± 3710	12,004 ± 2620	<0.001
VE/VCO_2_ slope	27.6 ± 4.8	23.4 ± 2.5	<0.001
VE/VCO_2_ slope (Z score)	−0.2 ± 1.4	−1.4 ± 0.7	<0.001
R.E.R.	1.15 ± 0.1	1.15 ± 0.1	NS

Data are expressed as mean ± SD. SBP: systolic blood pressure. HR: heart rate; VO_2_: oxygen uptake; AT: anaerobic threshold; CP: circulatory power; VE/VCO_2_ slope: relation between ventilation versus carbon dioxide production; R.E.R.: respiratory exchange ratio.

**Table 3:** Comparison between main clinical variables between HCM patients who did not experience (No Event Group) or experienced (Event Group) adverse events.

General Data	No Event Group (*n* = 46 Patients)	Event Group (*n* = 14 Patients)	*p*-Values
Age, years	15 ± 2	15 ± 2	NS
BSA, m^2^	1.7 ± 0.4	1.7 ± 0.3	NS
Male, %	36 (78)	8 (57)	NS
Age at diagnosis, years	9 ± 5	9 ± 3	NS
NYHA II, n (%)	7 (15)	8 (57)	<0.001
LVOT obstruction, n (%)	8 (17)	3 (14)	NS
ICD, n (%)	5 (11)	7 (50)	0.003
**SCD Risk Factors**			
NSVT, n (%)	3 (6)	3 (21)	NS
FH-SCD, n (%)	2 (4)	1 (7)	NS
MWT > 30 mm or Z score > 6, n (%)	8 (17)	4 (28)	NS
Unexplained syncope, n (%)	2 (4)	6 (43)	0.001
ABPRE, n (%)	10 (22)	8 (57)	0.016
**Echocardiographic Data**			
LVEDd, mm	42 ± 6	42 ± 4	NS
LAd, mm	35 ± 7	42 ± 8	0.006
LAd (Z-score)	1.9 ± 1.4	3.4 ± 1.4	0.003
MWT, mm	21±7	23 ± 7	NS
MWT (Z-score)	4.4 ± 1.4	5.1 ± 1.7	NS
LVOTG_max_, mm Hg	8 [9]	10 [9]	NS
LVEF, %	63 ± 5	59 ± 6	0.004

Data are expressed as mean ± SD, as absolute number of patients (% on total sample) or as median [25th–75th percentile]. NYHA: New York Heart Association; ICD: implantable cardioverter defibrillator; LVOT: left ventricular outflow tract; SCD: sudden cardiac death; NSVT: non-sustained ventricular tachycardia; FH: family history; ABPRE: abnormal blood pressure response at exercise; LVEDd: left ventricular end diastolic diameter; LAd: left atrial diameter; MWT: maximum wall thickness; LVOTGmax: maximal LV outflow tract gradient; LVEF: LV ejection fraction.

**Table 4:** Comparison between the main CPET variables between HCM patients who did not experience (No Event Group) or experienced (Event Group) adverse events.

CPET Data	No Event Group (*n* = 46 Patients)	Event Group (*n* = 14 Patients)	*p*-Values
Exercise time, minutes	10.3 ± 1.7	9.8 ± 1.5	NS
Peak workload, watts	125 ± 43	89 ± 36	0.007
Peak SBP, mm Hg	38 ± 26	25 ± 20	0.042
Peak HR, % of predicted	80 ± 11	74±11	NS
VO_2_ AT, mL/kg/min	20 ± 4	14 ± 8	<0.001
Peak VO_2_, mL/kg/min	30 ± 8	21±6	<0.001
Peak VO_2_, % of predicted	67 ± 16	50±15	0.001
Peak VO_2_ (Z-score)	−2.6 ± 1.1	−3.6±1.1	0.004
CP, mL/kg/min*mm Hg	4557 ± 1793	2603 ± 1044	<0.001
CP%, % of predicted*mm Hg	10,090 ± 3597	6174 ± 2246	<0.001
VE/VCO_2_ slope	27.0 ± 4.1	29.7 ± 6.1	0.050
VE/VCO_2_ slope (Z score)	−0.3 ± 1.2	0.1 ± 1.7	NS
R.E.R.	1.15 ± 0.1	1.14 ± 0.1	NS

Data are expressed as mean ± SD. SBP: systolic blood pressure. HR: heart rate; VO_2_: oxygen uptake; AT: anaerobic threshold; CP: circulatory power; VE/VCO_2_ slope: relation between ventilation versus carbon dioxide production; R.E.R.: respiratory exchange ratio.

**Table 5:** Significant univariate Cox proportional survival analysis according to the main variables for the pre-specified cardiovascular end-point.

	Composite Cardiovascular End-Point (*n* = 14 Events)		
	H.R. (95% C.I.)	*p*-Values	C-Index
Unexplained syncope, n (%)	2.58 (0.82–8.13)	0.108	0.61
LAd	1.07 (1.00–1.15)	0.041	0.58
LAd (Z-score)	1.79 (1.13–2.86)	0.014	0.69
LVEF, %	0.90 (0.82–0.99)	0.030	0.59
Peak VO_2_, mL/kg/min	0.92 (0.86–0.99)	0.034	0.66
Peak VO_2_, % of predicted	0.96 (0.93–0.99)	0.025	0.70
Peak VO_2_ (Z-score)	0.66 (0.41–1.06)	0.089	0.61
CP, mL/kg/min*mm Hg	0.999 (0.998–0.999)	0.031	0.69
CP%, % of predicted*mm Hg	0.997 (0.995–1.00)	0.021	0.71

H.R.: hazard ratio; C.I.: confidence interval. LAd: left atrial diameter; LVEF: LV ejection fraction; VO_2_: oxygen uptake; CP: circulatory power. Only variables with a statistical significance at 15% were included in the table.

## Data Availability

The datasets generated during and/or analysed during the current study are available from the corresponding author on reasonable request.
